# Sub-Diaphragmatic Abcess

**Published:** 1894-05-12

**Authors:** 


### SUB-DIAPHRAGMATIC ABCESS.

Two cases of subphrenic pyo-pneumothorax are recorded by Dr. Lee Dickinson.[1] In both cases there were tympanitic swellings in the upper part of the abdomen, with signs of disease at the base of the lung on the corresponding side. One was found *post-mortem* to be due to a localised collection of pus and gas from perforation of a gastric ulcer; the other was presumably of the same origin (or the perforation of a duodenal ulcer), but no ulcer was found at this operation, and as the patient recovered after drainage of the cavity no further examination was made. This result was highly satisfactory, as the subphrenic collection of pus and gas was very large, compressing the base of the lung, and of ten days' duration. In the second case a large collection of pus in the epigastrum was drained, and later an empyæma on the same side, and both cavities were contracting satisfactorily when the patient died of septic poisoning. A cicatrised ulcer was found on the stomach. Dr. Dickinson points out that when the perforation occurs in the stomach the pus and gas collect beneath the left wing of the diaphragm, limited on the right by the falciform ligament; if it occurs in the duodenum it is on the right of the falciform ligament under the left wing of the diaphragm. In a great many cases of perforation the peritoneal suppuration is limited. Out of sixty-one records of *post-mortems* at St. George's Hospital, in fifteen cases was the suppuration completely circumscribed, and in nine incompletely. After laparotomy even a subphrenic collection of pus may form, probably from the site of the earliest extravasation from the stomach not being thoroughly cleansed, and hence secondary suppuration in the chest may follow.

Vanlair[2] records a case of sub-diaphragmatic abscess in a child which presented in the epigastrum. It lay between the diaphragm and the liver and colon, and had opened into the latter. At first there was no tympanitic condition, and the origin of the suppuration was never determined, as fortunately the child recovered after drainage, though perforation of the diaphragm and pneumothorax were present. It is stated that only one other case has been recorded in childhood.

[1] The British Med. Journal, 1894, vol. i., p. 233. [2] Med. Chronicle, Jan., 1894; abstract of original paper in Rev. de Méd., July, 1893.

